# Supplementary material for: Working memory capacity of crows and monkeys arises from similar neuronal computations
Source: eLife. 2021 Dec 3;10:e72783. doi: 10.7554/eLife.72783 (PMC8660017; doi:10.7554/eLife.72783)
Supplement: Transparent reporting form [file elife-72783-transrepform1.pdf]

1st Floor
24 Hills Road
Cambridge CB2 1JP, UK

P 01223 855340
W elifesciences.org
T @elife

eLife's transparent reporting form

We encourage authors to provide detailed information *within their submission* to facilitate the interpretation and replication of experiments. Authors can upload supporting documentation to indicate the use of appropriate reporting guidelines for health-related research (see EQUATOR Network), life science research (see the BioSharing Information Resource), or the ARRIVE guidelines for reporting work involving animal research. Where applicable, authors should refer to any relevant reporting standards documents in this form.

If you have any questions, please consult our Journal Policies and/or contact us: editorial@elifesciences.org.

Sample-size estimation

- You should state whether an appropriate sample size was computed when the study was being designed
- You should state the statistical method of sample size computation and any required assumptions
- If no explicit power analysis was used, you should describe how you decided what sample (replicate) size (number) to use

Please outline where this information can be found within the submission (e.g., sections or figure legends), or explain why this information doesn't apply to your submission:

Statistical analysis was performed at the level of identifiable single cells. All identified single cell units were included for analysis. In total 362 neurons were analyzed, a typical sample size comparable to previous studies on the same subject (e.g. Buschman et al., 2011), details are given in the section 'Electrophysiological recordings'. The numbers of analyzed neurons are given throughout the results section and as part of the figures and figure captions. Analysis of each neuron was dependent on the amount of correct trials within a recording session. Further subdivisions of the sample ('neuronal subpopulations') and the motivation to subdivide is reported in the Methods section 'Population analysis' and accompanying Table 1.

Replicates

- You should report how often each experiment was performed
- You should include a definition of biological versus technical replication
- The data obtained should be provided and sufficient information should be provided to indicate the number of independent biological and/or technical replicates
- If you encountered any outliers, you should describe how these were handled
- Criteria for exclusion/inclusion of data should be clearly stated
- High-throughput sequence data should be uploaded before submission, with a private link for reviewers provided (these are available from both GEO and ArrayExpress)

Please outline where this information can be found within the submission (e.g., sections or figure legends), or explain why this information doesn't apply to your submission:

The data reported (both behavior and neurophysiology of multiple neurons) was replicated in 2 animals and 362 neurons (biological replication). Two animals is the standard minimum established by the field, as a compromise between the 3R principles and required inter animal replication. Detailed information is given in the materials and methods sections 'Subjects' and 'Electrophysiological recordings'.

eLIFE
1st Floor
24 Hills Road
Cambridge CB2 1JP, UK
P 01223 855340
W elifesciences.org
T @elife

Statistical reporting

- Statistical analysis methods should be described and justified
- Raw data should be presented in figures whenever informative to do so (typically when N per group is less than 10)
- For each experiment, you should identify the statistical tests used, exact values of N, definitions of center, methods of multiple test correction, and dispersion and precision measures (e.g., mean, median, SD, SEM, confidence intervals; and, for the major substantive results, a measure of effect size (e.g., Pearson's r, Cohen's d)
- Report exact p-values wherever possible alongside the summary statistics and 95% confidence intervals. These should be reported for all key questions and not only when the p-value is less than 0.05.

Please outline where this information can be found within the submission (e.g., sections or figure legends), or explain why this information doesn't apply to your submission:

Statistical analyses are described and justified in in the Methods in the sections 'Data analysis', 'Information about color identity', and 'Population analyses'.
Applied statistical tests and accompanying measures are reported in the results section, in line with the test result summary. Further detailed information about statistical tests is available in a supporting file.

(For large datasets, or papers with a very large number of statistical tests, you may upload a single table file with tests, Ns, etc., with reference to sections in the manuscript.)

Group allocation

- Indicate how samples were allocated into experimental groups (in the case of clinical studies, please specify allocation to treatment method); if randomization was used, please also state if restricted randomization was applied
- Indicate if masking was used during group allocation, data collection and/or data analysis

Please outline where this information can be found within the submission (e.g., sections or figure legends), or explain why this information doesn't apply to your submission:

Trial conditions (number and identity of displayed colors) were presented to the animal in pseudorandom order (trial conditions could repeat for a maximum of two consecutive trials) with randomly chosen color pairs. Details are reported in the Methods section 'Behavioral protocol'. Group allocation was not masked, as the animal was blind to the upcoming trial condition and allocation was performed automatically using Matlab.

Additional data files ("source data")

- We encourage you to upload relevant additional data files, such as numerical data that are represented as a graph in a figure, or as a summary table
- Where provided, these should be in the most useful format, and they can be uploaded as "Source data" files linked to a main figure or table
- Include model definition files including the full list of parameters used
- Include code used for data analysis (e.g., R, MatLab)
- Avoid stating that data files are "available upon request"

Please indicate the figures or tables for which source data files have been provided:

eLife Sciences Publications, Ltd is a limited liability non-profit non-stock corporation incorporated in the State of Delaware, USA, with company number 5030732, and is registered in the UK with company number FC030576 and branch number BR015634 at the address 1st Floor, 24 Hills Road, Cambridge CB2 1JP | August 2014

Details of the statistical tests are provided as a supporting file.
Source data and analysis code will be uploaded to a publicly accessible depository (gitlab). Should the reviewing process require access to additional source data and code beforehand, private access will be provided to the reviewers.
